# Book Reviews

**Published:** 1830-05

**Authors:** 


### CRITICAL ANALYSES.

Quæ laudanda forent, et quæ culpanda, vicissim

Illa, prius, cretâ; mox hæc, carbone, *notamus.*—PERSIUS.

*A Letter to* Sir HENRY HALFORD, Bart. K.C.H., *President of the Royal College of Physicians, &c., touching some Points of the Evidence and Observations of Counsel, on a Commission of Lunacy on Mr. Edward Davies.* By G. MAN BURROWS, M.D.—8vo. pp. 38. London: T. and G. Underwood, 1830.

THERE may, perhaps, be some difference of opinion as to the necessity for the publication of this letter. For our own parts, we think that Dr. BURROWS might safely have passed by the misrepresentations of newspapers, and the virulent attacks of a cross-examining advocate, with contemptuous silence. His character must be too well established in the estimation of his professional brethren to suffer aught from the garbled statements of the one, or the habitual abuse of the other, when any point is to be obtained which can tell in favor of the cause for which he is paid to plead. Dr. Burrows, however, may have felt that the public had a right to expect a refutation of the various attacks that have been made upon him respecting the evidence he gave upon the late commission *de lunatico inquirendo* on Mr. Edward Davies; and we confess it would have been difficult to withstand the temptation of giving publicity to so clear, so satisfactory, and so triumphant, an answer to his maligners, as that which is embodied in the present letter. To every impartial and disinterested man, it must be a source of much gratification to find that Dr. Burrows has not stept in one degree from the high station he has always maintained, either as a man of moral integrity, or as a physician of much more than ordinary ability. As we are mortified when we discover that our penetration has been deceived, and our confidence consequently misplaced, so are we rejoiced to find that he whom we have always respected comes forth with untainted reputation from the assaults of malevolence and misrepresentation.

We pass over the account which Dr. Burrows gives of the coarse and systematic abuse by which he has been persecuted, and come at once to a very important part of his address, namely, the principal charges made by Mr. Brougham when commenting upon his evidence.

In the first place, it was urged by Mr. Brougham that Dr. Burrows "betrayed more the feeling of a partisan than an impartial witness, attending to give unbiassed evidence; for he was always desiring to go on his own way, without interruption."* Now, it must be very evident that a witness in a cause may wish to go on "without interruption," not because he is either a partisan or is partial, but that his mind may not be distracted from the correct relation of the true and faithful details of the case he has to state. To this insinuative charge of Mr. Brougham's, Dr. Burrows replies that, in all commissions which he has ever attended, the commissioners have rather encouraged the medical witness to narrate his observations on the alleged lunatic, as being the best and shortest way of obtaining his evidence; and afterwards the court and counsel have examined and cross-examined him. He wished to pursue this course; but Sir Charles Wetherell, perhaps from an imperfect knowledge of the order of the facts he had to state, was, by putting questions relative to those subsequent, interrupting him, to the omission of important intervening facts. Dr. Burrows, therefore, begged permission to proceed in his own course; and in so doing, he merely followed that adopted by all the other medical witnesses.

Secondly, Mr. Brougham asserted that Dr. Burrows was mainly instrumental in sending Mr. Davies to his (Dr. Burrows') house at Clapham. This charge was unequivocally contradicted in evidence. It was clearly proved that Mr. Davies was sent to Clapham Retreat under the certificate of Mr. Lawrence and Dr. Blundell, and that Dr. Burrows was in nowise instrumental to his removal thither.

Thirdly, it was alleged by Mr. Brougham that Dr. Burrows put his name to a certificate which was to consign a fellow-citizen to a MADHOUSE, under the restraint of keepers, though, for ten days before, he had had no opportunity of knowing whether he was sane or insane. But how stands the fact? Why, it was deposed, on the oaths of Dr. Blundell and Mr. Lawrence, that the certificate of insanity which consigned Mr. Davies to a madhouse was signed by *them*, and *not* by Dr. Burrows. Mr. Brougham, indeed, chose to characterize, as a certificate of insanity by which Mr. Davies was to be sent to a *madhouse*, a note Dr. Burrows was directed to write to the master of the Fur-

* All the extracts from Dr. Burrows' evidence, or from Mr. Brougham's speech, are taken from the shorthand writer's notes, (Mr. Gurney's.)

nival's Inn coffeehouse, to show that the bearers of it were appointed to convey Mr. D. to his *own* house at Hornsey. If so gross a misstatement of the fact arose from carelessness and inattention to the evidence on the part of Mr. Brougham, it is highly discreditable to him; for surely an advocate, however earnestly he may wish for the successful issue of his cause, ought most carefully and deliberately to weigh every particle of the evidence adduced, before he ventures upon unsupported assertions, which are calculated to injure the moral and professional reputation of any of the parties to whom he may be opposed.*

There is one point of considerable importance upon which Dr. Burrows comments, and it is essential that the profession should be acquainted with what is required by the law upon the subject. It is quite a misconception to suppose that a medical certificate of insanity is required when a lunatic is placed *by his relations* under restraint in his own house, or is removed *by their orders* from his house to another abode, or from any place where he may have taken refuge, to his own house. "Nor has it ever been the practice, nor is it understood by the profession to be required by law, to give a certificate of insanity with a patient in any such case. But, if it be intended to remove a lunatic to a private house or lodgings, and to be there kept under the *exclusive charge and maintenance* of any one not a relative or of a committee, or to send him to an asylum or licensed house; in either of these cases a regular order must be signed by some responsible relation or friend, and after a separate examination of the patient by two medical practitioners, a form of certificate of insanity, prescribed by the Act of Parliament, must be signed by each of them. This constitutes a regular certificate, and must be transmitted with the patient."

Mr. Brougham further alleged that what occurred on Dr. Burrows' visit to Mr. Davies on the 31st of July, was not sufficient to warrant the conclusion that Mr. D. was insane on the 4th of August. Dr. Burrows, however, was prepared with irrefragable proofs of the continuance of the malady: but the legal advisers of Mr. Davies contended that these proofs were not admissible according to the rules of evidence. It is not for us to discuss the law which *prevents* a man from showing that he is perfectly innocent

* We know that Mr. Brougham has recently expressed his deep regret that any comments made by him should have been injurious to Dr. Burrows. Mr. Brougham avers that he acted strictly according to his instructions. —EDITOR.

of a charge which is brought against him, but it must appear to every reasonable mind that such a feature in the jurisprudence of the country calls loudly for amendment; and surely it is additionally incumbent upon an advocate, who knows that he can take advantage of so monstrous a legal technicality, to be doubly anxious not to advance any accusations which are not clearly substantiated by the evidence. To level unfounded accusations against a man who is prohibited from establishing their falsehood, is just as disgraceful as to inflict a blow upon him whose hands are manacled.

The last and most serious charge which was urged by Mr. Brougham against Dr. Burrows, was well calculated to have its intended influence with the jury. Mr. Brougham asserted that Dr. Burrows' evidence was given under the direct bias of interest; for, if Mr. Davies was found of sound mind, he would lose all the profits from retaining him in his asylum. It will scarcely be credited that Mr. Hobler, Mr. Davies' solicitor, knew Dr. Burrows had repeatedly requested that Mr. Davies might be removed from his asylum. When the day for commencing the inquiry was fixed, Mr. Davies himself declared he would not remove from Clapham Retreat, except force was used, unless he might be permitted to go, free from all control, to his house at Crouch hill. "His determination," says Dr. Burrows, "was approved by Mr. Hobler, and a strong appeal was made to me by both parties to *suffer* Mr. Davies to remain; and at length I gave a reluctant permission to his staying there till the inquiry was finished."

After all the labours of the commission, and the declamation of Mr. Brougham, it appears, from the following statement, that an erroneous verdict was returned. "The event has fully falsified the verdict. It appears that Mr. Davies has never, since that verdict was pronounced, evinced 'a sound mind,' nor been 'capable of managing himself and his affairs;' and, as the climax of this extraordinary case, he now acknowledges that he was, and still is, insane, and justifies those who have affirmed it; and has voluntarily placed himself under the care of two of the physicians who, on the inquiry, gave the strongest testimony of his existing insanity!"

We have now arrived at the termination of the very satisfactory replies which Dr. Burrows has made in answer to Mr. Brougham. Notwithstanding the provocation he has received, he indulges in no recriminative attacks upon his assailants, but with a calmness which few could have commanded under such irritating circumstances, he very liberally endeavours to account for the extreme cruelty with which he has been treated, in the following manner:

"However injurious the consequences of the very grave charges I have discussed, (charges which involve both my moral and professional character,) have proved to me, I am moved by no feeling of resentment against the learned counsel who has been the chief instrument in inflicting them. I cannot think it possible that a member of one liberal profession would make, in mere 'forensic wantonness,' accusations against a member of another profession which, from his intimate knowledge of public feeling on all subjects relative to the insane, he must be convinced were calculated to excite a powerful prejudice against him. I verily believe that Mr. Brougham did not exceed his instructions; for I can conceive in what spirit they were drawn up. I would rather in charity suppose he believed these instructions to be true. This might justify the course of cross-examination he pursued. But, if all the charges implied to the questions he put were denied or disproved on the oath of respectable witnesses, was it his duty, was it candid, was it even humane, to embody those charges in his speech, and urge them against me as if proven?"

We cannot quit this subject without mentioning a fact which must appear very extraordinary after the unexampled severity with which Mr. Brougham had treated Dr. Burrows in the course of Mr. Davies' case. Only a few days had elapsed after the termination of this inquiry, when Mr. Brougham was employed as counsel in a cause in the court of Delegates, in which Dr. Burrows had again to give his testimony. Upon this occasion a remarkable change was manifested in the tone and tenor of Mr. Brougham's address; for Dr. Burrows was mentioned by him as a highly respectable physician, whose opinions were not to be doubted.

As the statements which a man makes in defence of himself are liable to suspicion, we deem it necessary to remark that every part of Dr. Burrows' letter is confirmed by written documents, or the evidence of other persons. Enough has been said to show that a hostile party-feeling was particularly pointed at Dr. Burrows throughout the case of Mr. Davies. In a subsequent part of the letter, still more striking proofs of this fact are adduced. Every circumstance which occurred, and every comment made by counsel throughout the investigation, which was at all advantageous to Dr. Burrows, was omitted in some of the leading newspapers. In the report of Mr. Brougham's speech in "The Times" of the 21st of December, a sentence was inserted, very much to the discredit of Dr.

Burrows, which had never been uttered. To prevent the repetition of the calumny, Dr. B. addressed a very proper and temperate letter to the editor, to which he did not vouchsafe to make any reply. The inference is obvious.*

If, upon the present occasion, we have deviated from our usual custom of confining the review department of our Journal to subjects of practical interest, we are sure we shall stand excused when the motive is remembered. The duty we have performed has been gratifying to ourselves, and must be approved of by our readers. We have shown that the attempts which have been made to injure the reputation of Dr. Burrows have originated either from interested motives or misrepresentation. His ability in that particular branch of the profession to which he has of late years most especially devoted his attention, has been proved by his "Commentaries on Insanity," and is still more satisfactorily shown by the results of his practice. The proportion of the cases of insanity cured at the Clapham Retreat since its opening, is about three in four. The character and general management of this establishment may be inferred from the fact of its having been acknowledged, even by Mr. Davies' counsel, that there was no imputation on the Clapham Retreat. "During the residence of Mr. Davies in it, no similar establishment ever underwent such an ordeal; for, between the 3d of November and the 26th of December, he received above 250 visitors, most of whom went with a notion that he was not insane, and therefore looked with a jealous eye and a disposition ready to cavil at all they saw there." In every respect the triumph of Dr. Burrows is most complete, and we cannot doubt that he will meet with the cordial and zealous support of the profession and the public, as a mark of their indignation at the unworthy attempts that have been made to tarnish his reputation as a man and as a physician.

---

Since the above notice of Dr. Burrows' Letter to Sir Henry Halford was written, we have perused an article in the last Quarterly Review, which professes to be a review of Dr. Burrows' "Commentaries" and Dr. Haslam's "Observations on Madness." It is quite obvious that the purpose of this article was to afford the writer an opportunity of introducing his own views of Mr. Davies' case, and

* It has subsequently been admitted by the Editor of the Times, that Dr. Burrows "has been unfairly treated."—*Times*, April 1st.

of vituperating those physicians who gave their testimony upon it. The remarks that are made upon this case evince a determined and laboured effort to pervert the facts which were substantiated by evidence. The event has proved the weakness of the writer's judgment: for Mr. Davies *is now insane*,* and under the care of Sir George Tuthill and Dr. Haslam. When Dr. Burrows published his "Inquiry," he was lauded in the Quarterly Review as a sensible writer, and as an experienced and singularly successful practitioner in mental affections. The greater portion of this same "Inquiry" is embodied in his "Commentaries; and yet the latter work is now condemned in the Quarterly as a "wretched compilation," and it is discovered that Dr. Burrows lacks talent for the task he had imposed upon himself. Whence proceeds this extraordinary change of opinion? It cannot be supposed that the abilities of Dr. Burrows have declined with his years and extended experience. We deeply lament the explanation we have to offer of the total change in the opinions of the Quarterly Review; but our delicacy towards the dead would be excessive, if it induced us to withhold justice from the living. It is notorious that Dr. GOOCH was the author of the attack upon Dr. Burrows, and it is equally well known that he was the last man who ought to have ventured to criticise either the work or the conduct of Dr. Burrows; for Dr. Gooch was neither an unprejudiced nor a disinterested judge. He was biassed against Dr. Burrows by a pique under which he had long been smarting, and he was interested in marring the professional character of Dr. Burrows, because he was anxious to obtain for *himself* a reputation in the treatment of insanity.

> "Critics there are who other names deface,
> And fix their own with labour in their place."

Some apology may be found for Dr. Gooch. The vindictive attack upon Dr. Burrows and other physicians was written when the better feelings of his mind were annihilated by bodily pain and great mental irritability. For his friends no excuse can be urged: they should have suppressed the article altogether, instead of resting satisfied with depriving it of a small portion of its misplaced and uncharitable asperity. Dr. Gooch was justly respected for his talent, and it must be a source of sincere regret that he should have tarnished his fair name by this last of his literary labours. It would be unjust not to state that the very

* April 17th.

liberal, delicate, and witty pun upon Dr. Burrows' name, did not proceed from Dr. Gooch's pen: this exquisite specimen of vulgarity was added, after the original article had been revised and shorn of some of its foul proportions, by another person who polished it for publication.

It is lamented in the Quarterly Review, that "such a mass of trash" (as Dr. Burrows' "Commentaries,") should be in the hands of the English student of mental diseases, and go forth to foreign nations as a specimen of what the English mind is capable of effecting on such a subject." What a very different estimate has been formed of the talent of Dr. Burrows as a writer in foreign nations, may be gathered from the fact of his "Inquiry" having been translated into French and German. Of his "Commentaries" an American writer speaks in the following eulogistic terms: "It displays considerable research, industrious observation, correct and elevated feelings, and a spirit of candour and real love of truth. The author writes like an intelligent gentleman, a good physician, and pure moralist."* In all the English journals, Dr. Burrows' work was mentioned with the highest approbation.

---

*A Practical Essay on Stricture of the Rectum: illustrated by Cases, showing the Connexion of that Disease with Prolapsus of the Rectum, Irritation of the Lungs, Affections of the Urinary Organs and of the Uterus, Fistula, &c. To which is now added, some Practical Observations on Piles, and the Hæmorrhoidal Excrescence.* By Frederick Salmon, Senior Surgeon to the General Dispensary, Aldersgate street. *The Third Edition, very materially enlarged.*—8vo. pp. 272. Whittaker and Co. London, 1829.

It may fairly be inferred that there must be some merit in a work which has so quickly run on to a third edition. It is now but little more than two years since Mr. Salmon first published upon the important practical subjects to which he has devoted his attention. In our Journal for February 1828, we gave a brief analysis of the first edition of this work, and we shall now advert to those parts of it which we did not then notice, as well as to those which have been subsequently added.

In the preface to the present edition, the author informs us that, although the period which has elapsed since the publication of the last has been exceedingly limited, it has

* North American Med. and Surg. Journal, October 1829.

nevertheless afforded him an extended opportunity of practically applying the opinions he originally offered to the profession, and of witnessing their usefulness. He believes that a careful perusal of the various additions made to the present volume, and more particularly of those cases which evince beyond a doubt the connexion of affections of the uterus and the vagina with disease in the lower bowel, cannot fail of awakening the profession to the study of a malady which has hitherto been most superficially attended to.

Mr. Salmon had purposed to illustrate the various kinds of stricture by drawings, but unfortunately he intrusted a large quantity of morbid preparations, which he had been some years in collecting, to the care of a professional gentleman of reputed character, from whose possession the most valuable of those preparations were unaccountably lost, and he is consequently deprived not only of the opportunity of fulfilling his intention, but likewise of offering to the profession, as he had promised, a series of lectures upon the various diseases of the rectum. The loss Mr. Salmon laments does, indeed, appear unaccountable, and more especially as the "most valuable of the preparations" are missing: some scientific depredator must surely have them in safe custody.

Mr. Salmon first makes a few preliminary remarks on the anatomy of the rectum, and its relative position with the viscera in the pelvis, and then proceeds to enumerate the causes and different kinds of stricture, the morbid anatomy and situation of the disease. The symptoms and treatment of stricture are next described. The fifth chapter treats of the introduction and use of the bougie, of its formation, and also of gum-elastic, metallic, and medicated bougies. In chapter vi. the usual appearances which occur during the curative process of stricture are pointed out, and the proper manner of discontinuing the bougie is stated. As we have given, in the former Number of our Journal to which we have already alluded, an abstract of Mr. Salmon's opinions upon these subjects, we pass them over at present.

Upon the subject of dividing the stricture, Mr. S. is of opinion that, taking into consideration the danger consequent upon hemorrhage, the probability of inflammation, and the distressing irritation which arises from the necessity of keeping the divided portion of the gut separated during the healing process, the division of the stricture may be always accounted a dangerous, and in many cases an equally useless operation. There are few instances where it can be attempted with utility and safety.

"It may be performed, where the obstruction is sufficiently near to the orifice to admit of an examination of the disease being made with the finger;* indeed, unless this can be accomplished, I should not feel justified in attempting the operation, since there is not any safe criterion whereby to judge of the extent and nature of the contraction, or any guide to prevent our injuring blood-vessels of considerable magnitude, the hemorrhage from which might, under circumstances of local irritation, prove fatal.

"Even under the most favorable progress, the patient will find a difficulty in retaining the bougie in the bowel after it has been divided, which is essential to ensure the success of the operation.

"It may be necessary, in making these observations, to draw the reader's attention to the distinction between that kind of obstruction which is produced from the formation of adventitious bands, or septa in the rectum, and stricture of the gut itself; since, as I have before mentioned, the former may be freely divided, without any apprehension of danger.

"Having resolved upon the division of the part, we proceed with the operation in the following manner: The forefinger of the left hand being introduced into the rectum, to ascertain the most prominent parts of the stricture, and that no vessels of magnitude prevent its safe division, a broad probe-pointed bistoury, its side resting upon the finger, is to be passed fairly beyond the obstruction; the edge should then be everted, and the stricture divided in one or more parts, as may be necessary.† It will generally be requisite to divide the intestine in several bearings, and we should not be satisfied till we are able to introduce a bougie of the size of number nine or ten. I think this mode of division preferable to that of one deep incision; since we cannot with safety completely separate the muscular coat of the intestine, excepting towards the sacrum; and, even in this situation, there is considerable hazard in so doing." (P. 62.)

When the hemorrhage has ceased, the rectum is to be cleansed by an enema of tepid poppy water, and a portion of bougie, well oiled and covered with lint, or a plug entirely composed of the latter material, introduced, of sufficient size to distend the bowel. It is not necessary to pass this farther than through the part which has been divided. If practicable, this should remain in the rectum twenty-four hours, by which period discharge will generally have commenced; after which we may be satisfied that no union by adhesive inflammation will take place. "It is then to be

* "This, of course, does not apply to carcinomatous disease of the rectum."

† "Sir Astley Cooper's knife for hernia, made broader in the back than is common, is an excellent instrument to use for this purpose."

removed, and, after the bowel has been emptied by an enema, to be replaced by another plug, smeared over with simple ointment." This plan is to be adopted daily till the intestine is healed, which will be evinced by the absence of discharge upon the dressing. During the curative process, the smallest quantity of the most nutritious food is to be allowed; and after the first two days, a dose of castor oil is to be given. When the part is healed, a full-sized bougie must be introduced every third or fourth day, suffering it to remain in the bowel till the patient is desirous, from the irritation it excites, to have it removed. "If inflammation occur, or great local irritation ensue, the bougie must be immediately removed, and the necessary measures adopted, *with the utmost promptitude and decision.*" Mr. Salmon, however, is averse to this operation: he does not think it likely to be productive of that degree of success which may be considered a compensation for so hazardous and painful an expedient.

Upon the subject of carcinoma of the rectum, Mr. Salmon is very brief: he is apparently unacquainted with the operations of LISFRANC, who has removed cancerous portions of the lower part of the rectum in several instances, with success. A brief account of these cases is given in the Journal des Progrès des Sciences Médicales, tom. i. 1830, p. 266.

In the ninth chapter, the author offers some further remarks on various affections that may be considered as the necessary consequences, if not the immediate symptoms, of stricture of the rectum; and first of the *urinary organs.* We need only refer to the relative situation of the bladder and the rectum, to perceive how natural it is for the functions of the former to be more or less disturbed by stricture in the rectum. The constant efforts to relieve the bowels, by occasioning pressure upon the fundus of the bladder, speedily excites a corresponding irritation in this part, inducing a frequent desire to void the urine; while accumulations, collecting in the lower portion of the rectum, irritate the neck of the bladder more particularly, and promote spasmodic action to such a degree as even entirely to paralyse its functions: hence we may have either incontinence or retention of urine from the same source.

"The exciting cause remaining, nature will proceed to relieve herself by the formation of matter, either at the neck of the bladder or in the prostate gland; and, in extreme instances, disease will extend into the mucous coat of the bladder, and thence even into the kidneys themselves. A singular and interesting case of the two latter affections combined, the result of mechanical pressure, from a large tumor on the pelvis filled with hydatids, will be found in the sequel. In this instance it was somewhat remarkable that, although the urinary organs were considerably disturbed, and the patient died from matter forming in both the kidneys, the symptoms of obstruction in the rectum were not of sufficient importance to lead to any examination of the part. Though the passage through the bowel must have been greatly obstructed by the pressure of the tumor, there was not any difficulty of passing relief from the bowels: there was, however, a frequent desire so to do, and the stools were of small size, but in other respects they were healthy; yet the irritability of the urinary organs was so great, that the patient was obliged to pass his water every two or three hours, in doing which he experienced much difficulty and pain. I here suspected obstruction in the urethra, and in consequence introduced a bougie into that canal, which only aggravated his sufferings; yet I encountered no symptom of disease, save extreme tenderness at the prostatic part of the canal.

" The collection in the rectum before alluded to, aggravated by an enlarged and distended state of the colon, pressing upon the kidneys, deranges their function, giving rise to unhealthy secretion, whence may originate the formation of calculi; so, likewise, the irritation propagated to the canal of the urethra may promote either morbid discharges or the momentous malady of stricture." (P. 72.)

That these various effects may result from the same cause, is illustrated by several cases.

Chapter x. "Of affections of the Uterus and Vagina." Mr. Salmon has no doubt of the intimate connexion of many affections to which the uterus is liable, with irritation or obstruction in the rectum. For some years he has acted upon this principle in practice with the happiest results.

" One of the most frequent and distressing effects of stricture, is an enlarged or tender condition of the uterus. These are most afflicting cases, since we are not able to introduce instruments of any size, the enlargement of the uterus creating a partial obliteration of the cavity of the intestine: hence the bowels are never regular, and the voiding of any relief from them is always attended with more or less difficulty and pain.

" As the contraction in the rectum increases, the symptoms of the case become more and more urgent, till at last, in extreme instances, ulceration takes place, and fistulous communications are formed between the vagina and the rectum. These generally happen at the situation of the recto-vaginal septum, where the parts are very thinly separated by intervening cellular tissue. A more lamentable affection than this cannot easily be imagined: happily, however, it is proportionably rare; for, though we cannot do much towards the removal of the stricture, by strict attention to the constitutional and local treatment, we are generally enabled to keep it stationary. These fistulæ, however, sometimes occur, where the stricture itself, from which they result, has been overlooked either altogether, or till it has reached a very serious extent. The best surgery is in these cases productive of little or no benefit.

"Even in the early stages of stricture, we generally find the uterus sympathise with the rectum, and indistinct appearances arise, leading us to suspect incipient disease in the former organ, which disease will disappear upon the removal of stricture in the rectum. Not unfrequently, however, is the treatment directed to the uterus, considering it as the primary affected part.

"I have had many cases illustrative of this fact: I remember one in particular, the subject of which had for some months been under the care of several experienced practitioners for what was considered to be 'an enlarged and tender state of the cervix uteri.' The indications of disease in this part altogether disappeared, on the removal of a simple spasmodic stricture in the rectum. Here the effect of enemas was truly surprising: in the course of a fortnight the irritability of the bladder, which was considerable, and the frequent inclination to relieve the bowels, subsided. The patient had still, however, a degree of difficulty in accomplishing the latter, resulting from the enlargement of the uterus.

"I have known discharges from the vagina to subside upon the relief of stricture of the rectum; and I believe that many cases of irregularity in the menstrual discharges originate in an irritable or contracted state of the lower bowel. This position is extremely probable, when we reflect upon the contiguity of the parts; for, from any accumulation in the rectum, pressure is made upon the uterus, which is increased at every attempt to relieve the bowels, irritating the former organ and disturbing its functions. Furthermore, in those instances in which stricture has existed in the lower bowel for a protracted period, adhesions form between the posterior surface of the uterus and the rectum; which adhesions not only displace the womb, but, by preventing its natural ascent out of the pelvis during the period of pregnancy, may give rise to the more formidable evil of miscarriage. (P. 121.)

In proof of the practical application of these observations, some interesting cases are related. We give the following as a specimen of affection of the uterus connected with stricture of the intestine.

"Mrs. ——, age thirty-five. October 1824. Complained of violent bearing-down pains, and of pains in the groins, extending into the region of the bladder, the lower part of the back, and down the thighs. For many years past she had suffered from dysmenorrhœa; occasionally voiding, during the period of the menses, a solid fibrous-like substance, which was followed by an offensive discharge from the vagina, which lasted for several days: the latter symptom she commonly experienced to some trifling extent; she also at times had great difficulty in making water. Upon inquiring into the state of her bowels, I found they were irregular, being scantily relieved at intervals of several days apart, with difficulty and pain; latterly she had been annoyed by itching and heat around the orifice of the rectum. Her general health was disordered; she suffered from flatulence, a sense of distention, and pain in the stomach after eating; also from pain in the head, especially at the back part. I recommended the immediate examination of the rectum, to which my patient readily assented. Having followed my usual plans for one week, on the 23d of October I examined the bowel.

"Upon attempting to introduce my finger, I encountered a solid obstruction, which I cannot better describe than as a ridge extended across the orifice at its upper and inner portion: this not only prevented the natural dilatation of the part, but formed a permanent obstacle to the passing of the contents of the bowel. The part was exceedingly irritable, and the introduction even of my finger produced considerable uneasiness. The sphincter was remarkably broad, and extended to the depth of a full inch. By examining per vaginam, I ascertained that the uterus was enlarged, and tender to the touch; it also appeared lower in the vagina than is common. Judging from the symptoms of the case, the protracted period of my patient's annoyances, and the examination, I was apprehensive of incipient disease in the uterus; being, however, certain that, while the orifice of the rectum was in its present state, no permanent improvement could take place, and having, by the introduction of a small-sized rectum bougie, ascertained the existence of stricture at the sigmoid flexure of the colon, I recommended the division of the sphincter, so as to allow of the introduction of instruments of sufficient size to remove the contraction above; giving it as my opinion, that the irritability of the urinary organs, the discharge per vaginam, and the condition of the general health, were the result of the obstruction in the rectum. She unhesitatingly consented to follow any treatment considered advisable.

"Having adopted my usual plans for ten days or a fortnight, introducing the same bougie every third or fourth day, I removed a triangular portion of the sphincter, and inserted a plug of sufficient size to distend the part, and thus to prevent its reuniting by adhesive inflammation. The plug was removed twice every day, and the bowel kept free from collections by the use of an enema, till the wound was healed, which it was in the space of three weeks, when I began the introduction of Mr. White's bougies.

It is somewhat singular that, a day or two after the performance of the operation, all discharge ceased, and the bowels were daily relieved without medicine. In the course of three weeks, however, the discharge returned in an increased quantity; she likewise experienced some difficulty in making water. I persisted in my usual plans, introducing the bougie every fourth or fifth day for two months. The use of the instrument gave her no kind of uneasiness, excepting at the very summit of the gut. Her health improved, nor did she suffer so much either from the discharge or from pain during the period of the menses. By slow degrees I increased the size of the bougie, till I was able to pass the but five, when the discharge had ceased; her bowels also acted more regularly, and her general health was proportionably improved. I saw this patient every now and then during the following eight months, and introduced the same sized bougie; beyond which I could not advance, on account of the enlargement and displacement of the uterus.

"July 1829.—I have frequently seen this lady. Her general health is far better than it was prior to my attendance; she never has any discharge from the vagina, nor has she experienced the singular appearance during the period of menses more than half a dozen times. Her bowels, however, continue troublesome; she persists in the use of the enema, but has never attempted to pass the instrument." (P. 126.)

In the next chapter, the author treats of Distention and permanent Enlargement of the Colon. The surprising degree in which nature accommodates herself to diseased action, cannot be better exemplified than in the extensive enlargement which sometimes takes place in the colon, as a consequence of stricture either in the sigmoid flexure or in the rectum. Collections will from time to time take place, distending the bowel to an enormous size; which collections remaining for some time, impair the contractile power of the intestine, and thus create a permanent irregularity and a difficulty of voiding the contents of the bowel. In these instances, Mr. S. observes that it is often beneficial to wear a broad elastic belt round the body, applied sufficiently tight to afford support, but not to create unpleasant pressure.

"But distention of the colon is apt not only to derange the functions of other parts, but to cause appearances which, even under the closest examination, may be mistaken for a diseased condition of the structure of those parts. Any accumulation of food in the stomach, creating, from pressure, uneasiness and pain in the transverse arch of the colon, may be, and as I believe is, commonly treated as indigestion, or some derangement of the stomach. So, also, any material collection of feces in the upper parts of the ascending and descending portions of the bowel, is likely to be mistaken for an enlarged and diseased condition of the liver; while the continued pressure upon the diaphragm, causing more or less difficulty in respiration, may lead us to apprehend incipient affection of the lungs. Thus, in process of time, causes and effects become so intimately blended, that the most accurate examination is insufficient to discover the primarily affected part." (P. 165.)

In confirmation of these remarks, three interesting cases are related; but we must refer to the work itself for the details of them, as they are given at considerable length.

Chapter xii. "On Piles." This distressing disease may arise from various causes, but, according to Mr. Salmon, they all tend towards the same results, viz. distention of the minute vessels of the mucous coat of the rectum, and enlargement of the hemorrhoidal veins. Piles may therefore be produced by any circumstance, constitutional or mechanical, preternaturally exciting, or mechanically obstructing, the circulation in these particular parts.

"But another and extremely prevalent cause of the disease will be found in a contracted condition of some part of the rectum, which causes an accumulation of feculent matter in the bowel: this necessarily irritates it, and its mucous surface more especially; while the perpetual straining which accompanies the desire to relieve the bowels, (the result of the accumulation,) injects the minute vessels of the part, distends, and finally causes them to rupture; hence arises the hemorrhage generally experienced, more or less, by those who are subject to piles. Now, it is not always judicious suddenly to correct this effect; for, although it is a diseased action, it not unfrequently is the mode by which nature relieves herself, and it thus, perhaps, prevents the formation of a more serious disease. Through the same reason the hemorrhoidal veins may become distended, which seldom burst, but, enlarging, form permanent tumors in the rectum. This enlargement will continue to increase, provided the cause of the distention remains uncorrected, till, in extreme cases, the entire cavity of the intestine at its lower part will be nearly obliterated. I recollect an instance of this kind, in which, from the distention of the hemorrhoidal veins, the finger could not be introduced beyond the first joint; the patient was likewise the subject of fistula in ano.

"Another effect of irritation in the rectum is the coagulable lymph, which, from time to time, is thrown out upon the inner coat of the intestine, and between its muscular and mucous coats; which lymph, becoming organized, at last creates a mass of diseased superstructure, productive of intolerable pain." (P. 203.)

The brief comments Mr. Salmon offers on the medical treatment of piles, do not particularly claim our attention. With respect to the surgical removal of the hemorrhoidal excrescence, upon which much difference of opinion still exists, he is of opinion that, if the subject be dispassionately considered in all its bearings, the weight of evidence will greatly preponderate in favor of excision, in preference to the application of the ligature.

" To substantiate this position, let us suppose the instance of a surgeon being consulted by a patient suffering from an extreme case of the hemorrhoidal excrescence. He inquires into the state of the patient's general health, and ascertains that he has not any enlarged or otherwise diseased condition of the liver; no stricture in the rectum, nor any organic affection to which the formation of the excrescences may be reasonably attributed. He examines the part, and discovers one or more tumors originating in the rectum, and either protruding externally or being within the sphincter. On what is he to found his judgment as to the removal of the diseased parts, by excision or by ligature? I should say not so much upon the size of the tumors, and their extent of attachment to the rectum, as upon the condition of the hemorrhoidal veins. When the tumors are external to the orifice of the bowel, there cannot, I think, be a question as to the manner in which we ought to proceed; since, as we are able to quell any hemorrhage which may ensue, we have a perfect control over the only circumstance which (in this instance) militates against the operation by excision.

" In the removal by ligature, we shall have to encounter much local irritation, and not unfrequently severe constitutional disturbance, over which, when it is once excited, we have a very limited control. Provided there are several tumors (a common occurrence) we shall be necessitated to perform several operations. The application of the ligature is usually extremely painful, its operation tedious, and not unfrequently incomplete, either from the ligature getting loose, or by reason of the base of the tumor being left, which forms a nidus for the return of the disease. The treatment after the removal of the tumors is likewise protracted; and lastly, it is, I think, inapplicable where there is any material distention of the hemorrhoidal veins.

" Now, the removal of the excrescence by excision is more expeditious, it is more complete, the pain is less, as is the danger either of local inflammation or of constitutional disturbance; the parts heal more kindly; and, finally, when we are compelled to divide the enlarged hemorrhoidal veins, the probable danger from hemorrhage is not by any means so great as that which is to be apprehended from the constitutional and local disturbance which almost invariably follows the including of them in a ligature.

" The application of ligatures to veins is, I think, one of the most uncertain operations in surgery. I have so often seen fatal results follow their use, that I confess I am not a little prejudiced against the operation; and I believe that the failure of the removal of the hemorrhoidal excrescence by the ligature is often referrible to the injudicious manner in which the ligature is placed upon the enlarged hemorrhoidal veins.

" A reasonable objection may be advanced against the operation by excision, in the division of the mucous membrane of the part; but I would fearlessly ask, is the danger of inflammation from this cause greater than that which is likely to ensue from the application of a ligature to the same part? I should think not.

"So far as my experience has gone, I can only say that I have repeatedly performed the operation by excision with perfect success: occasionally I have had to encounter hemorrhage, but never to such an extent as to endanger the life of the patient, or, indeed, even to be a source of serious apprehension. I am inclined, therefore, to believe that, when bleeding ensues to any material extent, it is in those cases where the tumors are accompanied with an enlarged or otherwise diseased condition of the liver, with stricture of the bowel, or such an unhealthy condition of the constitution as may give rise to an hemorrhagic disposition in the vessels at the lower part of the alimentary canal; and, in the neglect of the due observance of any of which circumstances, not only the danger, but the unsuccessful issue, of *either* description of operation is very likely to originate.

"Prior to the removal of the hemorrhoidal excrescence by any kind of operation, we ought carefully to survey the various points to which I have alluded; and, above all, we ought to examine into the condition of the rectum; for, in the early stages of piles, where the disease is accompanied with any contraction of the bowel, we shall often be able to mitigate the former by the removal of the latter." (P. 209.)

Mr. Salmon has seen many instances which confirm this observation, and a few of them are detailed.

In the last chapter, a few remarks are made, and cases in point are related, of determination of blood to the head, occurring as a consequence of stricture of the rectum.

The practical opinions advocated by Mr. Salmon have evidently been formed from his personal observation, and as the subjects upon which he treats have received less consideration than many others of minor importance, his work must be considered an acceptable addition to the library of the surgical student.

---

*Observations on the Functional Disorders of the Kidneys, which give rise to the Formation of Urinary Calculi: with Remarks on their frequency in the County of Norfolk.* By WM. ENGLAND, M.D., Extraordinary Member of the Royal Medical and Royal Physical Societies of Edinburgh, and Member of the Physical Society of Guy's Hospital.—8vo. pp. 108. Underwood, London.

WE were induced to hope, from the preface to these observations, that Dr. ENGLAND had some new facts, or at least some very substantial theory, to offer, which would tend to explain the origin of calculous diseases in a more satisfactory manner than had hitherto been done. But we have been disappointed; for the greater part of the work is occupied by the repetition of opinions which have frequently been stated. We would not, however, refuse to Dr. England all claim to originality: he throws out a few new suggestions, but they are mostly crude and unsupported.

A brief sketch is first given of the different opinions that have been entertained respecting the origin of urinary calculi. The author contends that the attempt to explain their origin upon chemical reasoning, must be always attended with a complete failure:

"For, notwithstanding the rapid advances towards perfection which have distinguished that science during the last fifty years, it must be very obvious that the operations of chemical affinity must become altogether ineffective when we wish to make it exert any influence over vital laws: in fact, chemical agents, when they operate upon the animal economy, are altogether modified in their action by the laws of life.

"The application of chemical reasoning to pathology is in no case so erroneous as when an endeavour is made to account for the origin of calculous diseases upon chemical principles: the necessary consequence of directing our investigation in such a path, is that we are apt to overlook altogether the susceptibility which the kidney, as well as any other secreting gland, has of taking on certain morbid actions. As a proof of the tendency which an exclusive direction of our views to the play of chemical affinities has to render us blind to the consideration of the vital powers of a gland, that excellent practical chemist, Dr. Marcet, attributed the formation of urinary concretions to a separation and consolidation of certain ingredients contained in the urine; for he says that, 'independent of any specific agency of the urinary organs themselves, calculi are liable to form in any of the cavities to which the urine has access.'" (P. 11.)

We pass over many of the succeeding pages, not because we doubt the validity of the arguments contained in them, but because they are destitute of novelty. It is now universally admitted that, when the functions of the skin are deranged, and the digestive powers impaired, the quality of the urine is altered, and a tendency established to the production of calculous diseases.

Some interesting remarks are made on diet and habits of life, as exciting causes of renal disease. The frequency of calculous diseases in the county of Norfolk, in which the author resides, is attributed by him to a combination of causes; as the indigestible nature of the food of the working classes.

"No alimentary preparation is less capable of digestion than the

Norfolk dumpling, when eaten in the quantity in which it is consumed by the hard-working peasant: it gives great distention to the stomach, and being made of flour deprived of the bran, or cortical envelope of the grain, it has a natural tendency to induce constipation, when not combined with laxative adjuncts: it is, therefore, in this respect extremely inferior to oatmeal cakes, which have the property of inducing continually a regular action of the bowels by the mechanical stimulus of the particles of bran upon the nerves of the intestinal mucous membrane." (P. 70.)

The severe muscular exertion required in the use of the short-handled spade, which is generally employed by the Norfolk labourers, it is also supposed, may affect the functions of the kidneys.

"The relative situation of the kidneys with respect to the psoæ and quadrati lumborum muscles, and also their attachment to those muscles through the medium of adipose membrane and small blood-vessels, ought to be reflected upon when we investigate the obscure pathology of these organs. I am convinced that the great liability of the kidneys to be functionally disordered in consequence of over-exertion of the lumbar muscles, has been much overlooked.

"The important influence exerted upon the kidneys during the action of those muscles, is very evident: that very superior anatomist, the late Mr. Wilson, says, that 'branches from the lumbar arteries are sometimes found to enter the kidneys from behind; and I have also found branches from the renal arteries passing out from the substance of the kidneys, and forming communications with the vessels of the neighbouring parts.'" (P. 74.)

And, lastly, the geographical situation of the county of Norfolk is considered by Dr. England to be favorable to the production of calculous diseases.

"The geographical situation of the county of Norfolk renders its inhabitants more exposed to the impression of cold chilling winds than any other county of England; the whole of its north and east sides are directly acted upon by winds from those quarters; the former of which meets with no interruption to its progress from the icy deserts of Spitzbergen, and the latter passes over the flat districts of Holland and the north of Germany, to pour its unbroken and malignant force upon the exposed shore of this eastern county. From its peculiar position, and the absence of hills of sufficient elevation parallel with the shore, the air of this county is extremely cold in winter, and, during the early parts of the spring, vegetation is generally kept back by sharp easterly winds and a vast quantity of sleet." (P. 90.)

The frequency of urinary calculi in the county of Norfolk has been long known; but why such diseases should be more prevalent in this district than in many other parts of the country, is yet to be explained. The Norfolk dumpling may be very indigestible, the short spade a tool which requires great labour, and the inhabitants may be much exposed to cold and chilling winds; but in various other parts, where calculous diseases are comparatively rare, the labouring classes live upon equally indigestible food, work as hard, and are as much exposed to an ungenial climate. In our opinion, then, Dr. England has not satisfactorily shown why calculous diseases are so much more common in Norfolk than in many other districts.

The treatment of calculous diseases which Dr. England recommends differs but little from that of MASON GOOD, and many other practical writers upon the subject, whose works are well known.

---

*A Popular Summary of Vaccination, with Reference to its Efficacy, and probable Causes of Failure; as suggested by extensive Practical Experience.* By J. MARSHALL, Esq., Member of the Royal College of Surgeons in London, and District Vaccinator to the National Vaccine Establishment.—8vo. pp. 95. T. and G. Underwood, London, 1830.

THE object of this treatise is to bring into a narrow compass the principal causes of vaccine failure, in the hope of removing those doubts which have been too frequently thrown upon the practice of vaccination. The facts are supported by observation made during an extensive practice, under the direction and patronage of the National Vaccine Board. An arrangement has been adopted by Mr. MARSHALL, of classing the more prominent objects of interest under distinct heads, so that, by reference to the table of contents, the reader may at once become acquainted with all that is said upon any one point in the inquiry.

The subject of vaccination has been so frequently discussed in all its bearings, that we shall not enter into a regular analysis of each section of Mr. Marshall's summary. It may be sufficient to observe, that the points to which he has directed his attention are briefly yet satisfactorily treated. Judging from our own observation, we are inclined to believe that the confidence of the public in the protective power of vaccination is as great at the present moment as it has ever been. We very rarely hear any wish expressed for the inoculation of smallpox. That modified smallpox does occasionally occur after vaccination, is notorious; but this fact has ceased to cause the apprehensions it originally produced. With very rare exceptions, the modified variola is now known to be a mild and harmless disease, neither endangering the life nor personal appearance of the patient. There are some questions, however, in reference to vaccination, which are yet unsettled in the public mind, and which are perhaps not entirely set at rest amongst some of the members of the profession. To these we shall more especially refer.

That the vaccine lymph is not deteriorated by numerous transmissions through the human constitution, is still doubted by a few. Mr. Marshall says, that

"This fact is at once proved by the character of the vesicle being in every respect the same, after the lapse of thirty years, as that presented in the first instance, when immediately produced from the cow. Having been, from the year 1800, a governor of the original Vaccine Institution, and joint treasurer for some years, it has enabled me to add my testimony to the truth of this curious fact, and which is exemplified by the coloured engraving prefixed to the First Report of the Vaccine Institution, published in the year 1803, exhibiting progressive specimens of the vesicle in all its important stages, and which exactly accords with the graphical representations of Dr. Jenner. My appointment of district vaccinator to the National Vaccine Establishment has afforded the additional opportunity of minutely observing the repetition, in every essential point, in nearly three thousand cases. In the last Annual Report, dated March 2d, 1829, these remarks are most satisfactorily confirmed; 'for it does not appear to us to be weakened or deteriorated by transmission through any number of subjects in the course of any number of years.' Hence the virus of the vaccine vesicle has the same properties, as appears from the effects on the human constitution, whether it be generated by the cow or by man; and these properties are the same, however remote from the origin of the poison in the cow; and it may be continued, *ad infinitum*, from one person to another, without any necessity of recurring to the original matter of the cow." (P. 18.)

The period of taking the lymph for the purpose of vaccination, is a subject of infinite importance, and the regulations regarding it cannot be too deeply impressed on the attention of practitioners. We were once absolutely astounded at being very gravely informed by a practitioner of thirty years' standing, that *he* believed, and that he always had and always would act upon his opinion, that it signified nothing at what period "matter" was taken, whether at the fourth or fourteenth day of the vesicle, provided it could be obtained. Need we say that this gentleman had little or no confidence in the protective power of vaccination, or that his patients very frequently suffered from his obstinacy and ignorance. Mr. Marshall states that the lymph "cannot be used too early: as soon as the vesicle, even as early as on the fourth or fifth day, yields sufficient lymph to arm the lancet, it may be done with the surest effect; the usual time, and the latest recommended, is on the eighth day, prior to the full developement of the areola." We would rather take the lymph at the eighth day, in preference to an earlier period; not because we believe that the vesicle produced from vaccination at that period will be more perfect than if the lymph were taken before, but the public are so fully acquainted with the appearance of an eighth-day vesicle, from which lymph is usually taken, that they are dissatisfied, and remain apprehensive in future, if their children are vaccinated from an earlier vesicle, which to them appears imperfect. Upon a subject in which the feelings and anxieties of mothers are so deeply implicated, we may fairly yield a little even to their caprices, when the efficacy of our practice is not endangered. It must be remembered that, although Jenner recommends the ninth day, yet his practice was evidently guided by the state of the areola; for he interdicts "the use of the virus after the formation of the efflorescence around the pustule." The following remark from Mr. Marshall is worthy the attention of the practitioner.

"It seems however, that there is no general rule, either in grammar or life, without an exception; for, even on the eighth day, cases do now and then occur from some constitutional cause, where the vesicle is perfectly correct in all its phenomena, but with the surrounding inflammation at its full height, and even past, as displayed by the concentric circles. For the purpose of propagating vaccination, however, such cases ought invariably to be rejected." (P. 33.)

Some difference of opinion still exists as to the number of punctures that are necessary in the performance of the operation. Protection from the smallpox can be the only object, and we are not aware that any facts have arisen to show that this disease has occurred more frequently after vaccination where one puncture was made, than where there were several. The practice of depending upon one puncture, however, is deprecated by Mr. Marshall; and it is to be observed, that the Board of the National Vaccine Establishment have uniformly enforced the practice of making treble punctures in each arm, and they have also inculcated the absolute necessity of leaving one or two vesicles at least entire. We have heard several surgeons express their fears of violent inflammation, if several punctures were made. Such apprehensions our own observation taught us were groundless, and we find Mr. Marshall does not participate in them.

"It affords a gratifying consolation to be able to remark, notwithstanding the free manner of operating generally adopted, that only a single case has occurred of erysipelatous inflammation, extending from the shoulder to the elbow, after the twelfth day, which readily yield to a saturnine lotion. The arm thus affected had four vesicles, the other three, with the areola of the usual character and dimensions, marked by the concentric circles; whence an inference was drawn, that the inflammation had been effected either by accidental pressure or external injury. If it had arisen from a constitutional cause, both arms must have been equally affected, therefore it did not constitute a case of erysipelas, but mere local inflammation. Dr. Jenner mentions three instances of erysipelatous inflammation appearing on the vaccinated arm, which, by the application of mercurial ointment, subsided without giving much trouble." (P. 45.)

They who still require information upon the subject of vaccination, will do well to consult the brief volume we have just noticed. From being the district vaccinator to the National Vaccine Establishment, Mr. Marshall has extensive experience of his subject, which is the best claim to the attention of the profession.

---

*A Vade-Mecum of Morbid Anatomy, Medical and Chirurgical; with Pathological Observations and Symptoms. Illustrated by upwards of* 250 *Drawings.*—Burgess and Hill, London, 1830.

With whatever zeal practitioners in general may be inclined to prosecute every useful branch of professional information, it can fall to the lot of but few to command either the requisite time or opportunities for acquiring a competent knowledge of morbid anatomy. It is not the inspection of a few insulated cases that can impart this knowledge; months, and even years, of laborious investigation must be expended before we can obtain the tact of distinguishing with certainty even the appearances presented on dissection, after diseases of the most ordinary kind; while those alterations of structure which are produced by maladies of more rare occurrence, can in general be known only to those who enjoy unusual opportunities, and who possess unwearied diligence. Such a work as the present must therefore be a valuable acquisition to the majority of medical practitioners; for, although it is very true that neither graphic illustrations nor verbal descriptions can convey a perfect knowledge either of natural or morbid anatomy, they will certainly assist the memory in retaining what has once been practically learnt, and, if attentively studied, will impart a very useful degree of preparatory information of those appearances which have not yet been presented to the observation.

The objects which the author of this Vade-mecum has most zealously endeavoured to achieve, are to furnish a concise and faithful transcript of the labours of all the eminent morbid anatomical writers, and to illustrate the productions of his more elaborate predecessors. He has not, however, confined his task merely to transcribing and illustrating what others have seen and written; but he has blended in the work some account of what he has himself observed, during many years of close application to the subject. The work contains observations on, with illustrations of, the changes of structure found in the brain, thoracic, abdominal, and pelvic viscera, and of the organs of generation in both sexes. It likewise gives a brief sketch of the pathological symptoms by which we judge of disease during life, and a true description of the morbid changes that are exhibited after death. The sketches have been taken either from diseased parts in the author's museum or from recent dissection.

It forms no part of the plan of the work to give cases, or to introduce all the strange appearances that are occasionally found after death. A brief statement of symptoms and morbid phenomena in diseases the most likely to fall under the daily notice of the practitioner, has been wisely preferred to hypothesis, and to rare or doubtful subjects.

A regular analysis of a work, of which each page is occupied by a different subject, would be impossible; but we give the following extract as a specimen of the manner in which the whole is executed.

"Plate xiv. This plate represents acute and chronic inflammation of the œsophagus, and its effects. Fig. 1. Acute inflammation of the mucous tissue of the œsophagus. 2. Fibrine deposited in the œsophagus: it was moulded to the caliber of the tube, and slightly adhered to its mucous tissue. 3. An enlarged state of the mucous glands situated in the œsophagus. 4. Extensive ulceration of the mucous follicles of the œsophagus. Symp. *Inflammation of the Œsophagus.* Burning sensation on taking food; pain in the neck, particularly in the side, which is increased on pressure; dryness of the fauces; regurgitation of the food through the nostrils. *Ulceration.* An uneasy sensation in some part of the œsophagus, which is aggravated after eating, or after drinking ardent spirits; a lancinating or burning sensation in one or more parts of the tube; slight cough, cardialgia, repeated efforts to clear the throat of a tough dirty-coloured matter."

The morbid anatomy of all the organs of the body is thus illustrated. The symptomatology of the various diseases, of course, forms but a secondary object of the work; but the author has not failed to render his concise account of the leading symptoms very useful for the student.

We recommend this Vade-mecum of Morbid Anatomy to the attention of the profession; it will be valuable both to the junior and senior members, either for deliberate study or occasional reference. Although a considerable expense must have been incurred in getting it up, the price is extremely moderate. The publisher has doubtless calculated upon an extensive sale, and we believe he will not be disappointed.

